# Diagnostic difficulties in polymyalgia rheumatica cases with normal erythrocyte sedimentation rate and C-reactive protein values

**DOI:** 10.1097/MD.0000000000035385

**Published:** 2023-09-29

**Authors:** Mete Kara, Gülay Alp, Ali Murat Koç

**Affiliations:** a University of Health Sciences Izmir Bozyaka Research and Training Hospital, Rheumatology Department, Izmir, Turkey; b Izmir Katip Celebi University Izmir Atatürk Research and Training Hospital, Rheumatology Department, Izmir, Turkey; c Izmir Katip Celebi University Izmir Atatürk Research and Training Hospital, Department of Radiology, Izmir, Turkey.

**Keywords:** acute phase reactants, C-reactive protein, erythrocyte sedimentation rate, polymyalgia rheumatica, serum amyloid A

## Abstract

Polymyalgia Rheumatica (PMR) is an inflammatory disease which does not have specific diagnostic tests or pathological symptoms and is identified based on clinical characteristics. Among acute phase reactants (APR), the erythrocyte sedimentation rate (ESR) and C-Reactive Protein (CRP) are laboratory findings used in diagnosis and follow-up. In this study, it was aimed to determine the incidence of normal ESH and CRP in patients diagnosed with PMR and identify the distinguishing characteristics of these patients. PMR patients who were clinically diagnosed at a single center were reviewed. After the presence of bursitis was demonstrated with ultrasonography in patients with normal ESR and CRP rates, they were accepted to have PMR. Among all 54 patients (63% female), ESR and CRP values were normal in 8 patients (14%), and serum amyloid A (SAA) was determined to be elevated in all these patients. In the comparisons of the groups with normal and high levels of ESR and CRP, it was found that the group with normal ESR and CRP values had a younger age of diagnosis (*P* = .027), a longer symptom duration (*P* < .001), and a lower comorbidity rate (*P* = .010). PMR patients can have normal ESR and CRP values at the time of their diagnosis. While bursitis can be demonstrated with ultrasonography in patients who are clinically evaluated to have PMR, APRs such as SAA other than ESR and CRP can also be used.

## 1. Introduction

Polymyalgia rheumatica (PMR) is the most frequently encountered rheumatic disease in individuals over the age of fifty that is characterized by inflammatory pain in the proximal parts of the extremities and the body and elevated inflammatory parameters. Because it does not have gold standard diagnostic tests and pathological findings, PMR is diagnosed based on its clinical characteristics.

The initial diagnosis can be confirmed by the observation of a noticeable response to low-dose prednisolone in a week and careful monitoring to exclude an alternative diagnosis. Patients presenting with PMR symptoms should be monitored carefully because they can be diagnosed with diseases such as rheumatoid arthritis, remitting seronegative symmetrical synovitis with pitting edema syndrome, or malignancies in their follow-ups.^[1]^

The typical clinical presentation of the condition involves the persistence of morning stiffness for longer than 45 minutes, a symptom duration longer than 2 weeks, and elevated values of erythrocyte sedimentation rate (ESR) and C-reactive protein (CRP).^[2]^ The Provisional 2012 European League against Rheumatism (EULAR) and the American College of Rheumatology (ACR) classification criteria also include abnormal ESR values and CRP concentrations among the basic criteria for the diagnosis of PMR.^[3]^

Regarding acute phase reactant (APR) parameters in PMR patients, the most prevalently studied topic is ESR. High ESR levels were found to be associated with more relapse, lower rates of discontinuation of steroid treatment, and prolonged durations of steroid usage.^[4]^ Although ESR is very important for the diagnosis and follow-up of PMR, the ESR values of 7% to 22% of patients at the time of diagnosis may be normal. In up to 20% of patients, ESR may be below 40 mm/h. These patients usually have high CRP values. They also display fewer systemic symptoms, a milder clinical picture, and lower rates of anemia.^[5,6]^

There are contradictory reports on the view that CRP concentrations are more useful than ESR in the diagnosis of PMR. Nonetheless, results on the superior status of CRP to ESR in the initial diagnosis of PMR are uncertain.^[7]^ CRP can be used in follow-up to monitor disease activity because ESR can be affected by several physiological conditions including age, sex, and pregnancy.^[8]^ High ESR and CRP values not only help the diagnosis process but also are important in the assessment of disease activity and because of their negative predictive value. The diagnosis of PMR includes some difficulties because it can show heterogeneous clinical findings, an atypical onset, and normal APR values.^[6]^

Considering such a heterogeneous set of conditions, in our daily practice, we encounter patients who have normal ESR and CRP values. In this study, it was aimed to determine the prevalence of PMR patients with normal ESR and CRP values, find the differences of these patients, if any, from those with elevated ESR and/or CRP values, and identify the factors that can assist the diagnosis in this patient group.

## 2. Method

### 2.1. Design

This retrospective study, which was conducted between January 2020 and January 2022, included patients who were accepted as PMR according to the 2012 Provisional Polymyalgia Rheumatica Classification Criteria by the ACR/EULAR.

Because PMR has a highly heterogeneous clinical picture and can be confused with many other diseases, patients who had been followed up for at least 1 year were included. Patients whose diagnoses changed during their follow-ups were excluded. Moreover, patients who had previously been diagnosed with RA, GCA, or another inflammatory rheumatic disease requiring the use of disease-modifying anti-rheumatic drugs (DMARD) and those who were using steroids for other reasons were excluded.

The age, sex, and comorbidity characteristics of the patients, their smoking status, clinical and laboratory characteristics, treatment regimens, and information on those who developed PMR exacerbation or GCA during their follow-ups were obtained from electronic patient records.

### 2.2. Laboratory analyses

CRP was measured by immunoturbidimetry where CRP ≥ 0.5 mg/dL (≥5 mg/L) was considered abnormal. ESR was measured using the Westergren method where ESR > 25 mm/h was considered abnormal. For ESR, the 30-minutes automated version of the Westergren method was used. In this method, ESR is measured after 30-minutes, and the hourly ESR value is estimated using a certain algorithm. While this automated method saves time, it also has a very high degree of agreement with the manual 1-h Westergren method.^[9]^ ERS values above half of the numerical value of age in years plus ten in women and those above half of the numerical value of age in years in men were considered elevated. Serum amyloid A (SAA) values were analyzed using the enzyme-linked immunosorbent assay (ELISA) method.

### 2.3. Radiological evaluation

Ultrasonography (USG) was performed in all patients with normal CRP and/or ESR values. PMR classification criteria are based on a USG scoring algorithm that includes the presence of subdeltoid bursitis, biceps tenosynovitis, and/or glenohumeral synovitis in at least one shoulder, biceps tenosynovitis or glenohumeral synovitis in both shoulders for trochanteric bursitis, or subdeltoid bursitis in at least one hip.^[3]^ USG was performed by an experienced radiologist in all patients.

To detect and exclude other possible etiologies such as adhesive capsulitis and impingement in the differential diagnosis of PMR, MRI was performed in all patients with normal CRP. In one patient, positron emission tomography - computed tomography (PET/CT) was requested to exclude the presence of malignancies.

USG and MRI are practical and equally effective in confirming the presence of bursitis in PMR patients. We confirmed the presence of bursitis in all patients with normal ESR and CRP values in our study using USG, whereas we also used MRI in 5 patients and PET/CT in 1 patient for confirming the diagnosis of PMR.

### 2.4. Statistics

Descriptive statistics [mean (SD), median (IQR), or n (%) as appropriate] were used in the analyses, and differences between patients with normal CRP and/or ESR values and those with elevated values were tested using Fisher’s exact test for the categorical data, *t* test for the normally distributed data, and the Wilcoxon test for the non-normally distributed data. For all variables, normality assumptions were tested using the Shapiro–Wilk test. We compared the clinical characteristics of the PMR patients who had normal ESR and CRP values to those of the patients with elevated markers at the time of their diagnosis. We used Fisher’s exact test to compare the categorical variables between the patients with normal ESR and CRP at the time of their diagnosis and those with elevated values in either test. The patients were categorized into groups of ESR ≥ 40 mm/h and ESR < 40 mm/h and groups of CRP ≥ 5 mg/L and CRP < 5 mg/L, the categorical variables were compared using chi-squared tests, and the continuous variables were compared using non-parametric tests.

### 2.5. Ethical approval

Ethics committee approval for this study was received locally from the University of Health Sciences Izmir Bozyaka Research and Training Hospital (decision date: 10.11.2021, decision no: 187). The study was conducted in compliance with the principles of the Declaration of Helsinki.

## 3. Results

Among the 54 patients included, 34 (63%) were female, the mean age of all patients was 62.2 ± 6.7 years, and the median duration of symptoms in the patients was 5 months.^[4]^ The median ESR, CRP, and SAA values of the patients were respectively 54 (97), 12.4 (23), and 32 (25). SAA values were high in all twenty patients in whom they were checked. At least one comorbidity was observed in 77.7% of the patients. For radiological examinations to confirm the diagnosis, USG was used in 13 patients, MRI was used in 8 patients, PET-CT was used in 1 patient, and bursitis was found in all radiological examinations. Among the patients with normal ESR and CRP values, 8 were examined using USG, 5 were examined using MRI, and 1 was examined using PET-CT. Moreover, among those with high ESR and/or CRP values, 5 were examined using USG, and 3 were examined using MRI. During a follow-up of approximately 2 years, disease exacerbation developed in 15 patients, and GCA developed in 3 patients.

The other clinical characteristics of the patients are presented in Table 1.

**Table 1:** Baseline characteristics of all patients.

Variables	n:54
Age, years (SD)	62.2 (6.7)
Age at diagnosis, years (SD)	60.2 (6.7)
Follow-up duration, months (SD)	22.5 (6.6)
Sex, female, n (%)	34 (63)
Symptom duration, months, median (IQR)	3.5 (3)
Smoking status, n (%)	23 (42.6)
Bilateral shoulder pain/stiffness, n (%)	51 (94.4)
Neck pain, n (%)	41 (75.9)
Bilateral hip pain/stiffness, n (%)	41 (75.9)
Peripheral arthritis, n (%)	2 (3.7)
Systemic symptoms, n (%)	39 (72.2)
Elevated ESR, n (%)	39 (72.2)
Elevated CRP, n (%)	45 (83.3)
Elevated ESR and/or CRP, n (%)	46 (85.2)
ESR in mm/h, median (IQR)	59 (49)
CRP in mg/L, median (IQR)	16.2 (38)
Platelets, median (IQR)	302 (95)
Anemia, n (%)	26 (48.1)
SAA, median (IQR)	36 (26)
Rheumatoid factor present, n (%)	3 (5.6)
Anti-citrullinated C-peptide present, n (%)	1 (1.9)
At least one comorbidity, n (%)	42 (77.7)
Diabetes mellitus, n (%)	12 (22.2)
Hypertension, n (%)	11 (20.4)
Thyroid disease, n (%)	2 (3.7)
Ischemic heart disease, n (%)	8 (14.8)
COPD, n (%)	4 (7.4)
Malignancy, n (%)	3 (5.6)
Others, n (%)	2 (3.7)
Starting oral prednisone mg, median (IQR)	20 (0)
Methotrexate, n (%)	27 (50)
Azathioprine, n (%)	4 (7.4)
Tocilizumab, n (%)	3 (5.6)
Patients with exacerbations, n (%)	15 (27.8)
Giant cell arteritis, n (%)	3 (5.6)

COPD = chronic obstructive pulmonary disease, CRP = C-reactive protein, ESR = erythrocyte sedimentation rate, SAA = serum amyloid A.

While there were 15 patients without elevated ESR values, there were 9 patients without elevated CRP values. Eight (14.8%) patients had normal ESR and CRP values, and 46 (85.2%) patients had elevated ESR and/or CRP values. The patients with normal APR values had a longer median symptom duration before diagnosis (6 vs 3 months; *P* < .001) and a younger age at diagnosis (61.8 vs 68.5; *P* = .027). Fewer patients with normal APR values had systemic symptoms at diagnosis (37.5% vs 78.3%; *P* = .030), and these patients also had lower comorbidity rates (37.5% vs 89.8% *P* = .010). However, no significant differences were found in terms of the presence of peripheral arthritis, bilateral shoulder pain/stiffness, neck pain, bilateral hip pain/stiffness, rheumatoid factor (RF), or anti-citrullinated C-peptide (anti-CCP) values. The median platelet counts and the median SAA levels of the groups were found to be similar. There was no significant difference between the groups in terms of their starting dose of oral steroid treatment. The baseline characteristics of the patients with normal APR values and those with elevated APR values are presented in Table 2.

**Table 2:** Baseline characteristics of patients with normal and those with elevated ESR and CRP values.

Variables	Normal ESR and CRP (n = 8; 14.8%)	Elevated ESR and/or CRP (n = 46; 85.2%)	*P*
Age at diagnosis, years (SD)	61.8 (8.3)	68.5 (7.5)	**.027**
Sex, female, n (%)	7 (87.5)	27 (58.7)	.234
Symptom duration, months, median (IQR)	6 (1)	3 (1)	**<.001**
Smoking status, n (%)	1 (12.5)	22 (47.8)	.119
Bilateral shoulder pain/stiffness, n (%)	8 (100)	43 (93.5)	1
Neck pain, n (%)	2 (25)	11 (23.9)	1
Bilateral hip pain/stiffness, n (%)	5 (62.5)	36 (78.3)	.382
Peripheral arthritis, n (%)	0 (0)	2 (4.3)	1
Systemic symptoms, n (%)	3 (37.5)	36 (78.3)	**.030**
ESR in mm/h, median (IQR)	28 (4)	75 (31)	
CRP in mg/L, median (IQR)	3.9 (1)	34 (77)	
Platelets, median (IQR)	288 (103)	315 (85)	.619
Anemia, n (%)	3 (37.5)	23 (50)	.706
SAA, median (IQR)	31 (20)	37.5 (53)	.675
Rheumatoid factor present, n (%)	1 (12.5)	2 (4.3)	.388
At least one comorbidity, median (IQR)	3 (37.5)	34 (89.8)	**.010**
Starting oral prednisone mg (IQR)	18.75 (5)	20.1 (5)	.317
Oral prednisone only, n (%)	3 (37.5)	20 (43.5)	1
Steroid tapering treatment	5 (62.5)	25 (54.3)	.720
Methotrexate, n (%)	4 (50)	23 (50)	1
Azathioprine, n (%)	1 (12.5)	3 (6.5)	.484
Patients with exacerbations, n (%)	0 (0)	15 (32.6)	.089
Giant cell arteritis, n (%)	0 (0)	3 (6.5)	1

CRP = C-reactive protein, ESR = erythrocyte sedimentation rate, SAA = serum amyloid A.

Bold indicates significant (*P* < .05).

In our study, we used steroid tapering in 30 patients in total. Among these 30 patients, we used methotrexate (mtx) in 4 patients and azathioprine in 1 patient in the group with normal ESR and CRP values, while we used mtx in 23 patients (50%) and azathioprine in 3 patients (6.5%) in the group with high ESR and/or CRP values. Additionally, no significant differences in exacerbations per patient were found between the groups. During follow-up, no differences were found in the proportions of the patients with an additional diagnosis of GCA between the groups.

In the comparisons of the patients after their categorization into ESR < 40 mm/h and ESR ≥ 40 mm/h groups, no significant difference was found in terms of age at diagnosis or systemic symptom presence. The duration of symptoms and SAA values were similar between the two groups, whereas the ESR ≥ 40 group had significantly higher rates of PMR exacerbation and elevated CRP rates (respectively, *P* = .046 and *P* = .014).

In the comparisons of the patients after their categorization into CRP < 5 mg/L and CRP ≥ 5 mg/L groups, while there was no significant difference in terms of age at diagnosis between the two groups, their symptom durations, presence of systemic symptoms, and presence of comorbidities were different. Additionally, the elevated CRP group had significantly higher rates of ESR elevation and PMR exacerbation (respectively, *P* = .049 and *P* = .003). Other characteristics of these groups are shown in Table 3.

**Table 3:** Comparison of characteristics of patient groups with ESR < 40 mm/h and ESR ≥ 40 mm/h and patient groups with CRP < 5 mg/L and CRP ≥ 5 mg/L.

Variables	ESR < 40 mm/h (n = 10; 18.5%)	ESR ≥ 40 mm/h (n = 44; 81.4%)	*P*	CRP < 5 mg/L, (n = 9; 16.7%)	CRP ≥ 5 mg/L (n = 45; 83.3%)	*P*
Age at diagnosis, years, mean (SD)	57 (4)	63.5 (13)	.927	62.5 (15)	68.5 (10)	.325
Sex, female, n (%)	8 (80)	26 (59.1)	.291	8 (88.9)	26 (57.8)	.131
Symptom duration, months, median (IQR)	6.5 (1)	3.5 (1)	**.001**	6 (2)	3 (1)	**.003**
Smoking status, n (%)	2 (20)	21 (47.7)	.161	1 (11.1)	22 (48.9)	.062
Bilateral shoulder pain, n (%)	9 (90)	43 (95.5)	.466	9 (100)	42 (93.3)	1
Neck pain, n (%)	2 (20)	11 (25)	1	3 (33.3)	10 (22.2)	.670
Bilateral hip pain, n (%)	7 (70)	34 (77.3)	.689	5 (55.6)	36 (80)	.195
Peripheral arthritis, n (%)	0 (0)	2 (4.5)	1	0 (0)	2 (4.4)	1
Systemic symptoms, n (%)	5 (50)	34 (77.3)	.119	3 (33.3)	36 (80)	**.010**
Platelets, median (IQR)	245 (167)	290 (93)	.825	301 (76)	303 (103)	.903
Anemia, n (%)	4 (40)	22 (50)	.730	3 (33.3)	23 (51.1)	.470
SAA, median (IQR)	39 (26)	29 (14)	.415	34 (18)	36 (53)	1
Rheumatoid factor present, n (%)	2 (20)	1 (2.3)	.085	1 (11.1)	2 (4.4)	.428
At least one comorbidity, median (IQR)	5 (50)	37 (84.1)	.33	4 (44.4)	38 (84.4)	**0.019**
Starting oral prednisone mg, median (IQR)	17.5 (5)	20 (0)	.413	20 (3)	20 (0)	.367
Oral prednisone only, n (%)	5 (50)	18 (40.9)	.728	3 (33.3)	20 (44.4)	.717
Steroid tapering treatment, n (%)	5 (50)	25 (56.8)	.736	6 (66.7)	24 (53.3)	.715
Methotrexate, n (%)	4 (40)	23 (52.3)	.728	5 (55.6)	22 (48.9)	1
Azathioprine, n (%)	1 (10)	3 (6.8)	.571	1 (11.1)	3 (6.7)	.529
Patients with exacerbations, n (%)	0 (0)	15 (34.1)	**.046**	0 (0)	15 (33.3)	**.049**
Giant cell arteritis, n (%)	0 (0)	3 (6.8)	1	0 (0)	3 (6.7)	1
CRP in mg/L, median (IQR)	3.25 (3)	23.5 (59)	**.014**			
ESR in mm/h, median (IQR)				28 (5)	76 (30)	**.003**

CRP = C-reactive protein, ESR = erythrocyte sedimentation rate, SAA = serum amyloid A.

Bold indicates significant (*P* < .05).

## 4. Discussion

As seen in our study, some PMR patients can present with normal ESR and CRP values. These patients are younger than those who have elevated ESR and/or CRP levels, and they have a longer duration of symptoms and fewer comorbidities. Radiological methods can be utilized in these patients who have a clinical suspicion of PMR but no confirmation by laboratory data. In our study, the presence of bursitis was demonstrated by USG, MRI, or PET-CT in all patients who had normal ESR and CRP values at the time of their diagnosis. Even though their ESR and CRP values were normal, SAA values were found to be elevated in all these patients.

Approximately half of PMR patients display systemic symptoms such as low-grade fever, fatigue, and loss of appetite.^[11]^ In our study, we found the prevalence of systemic symptoms as 72%. Patients with normal ESR values are expected to have fewer systemic symptoms such as fever, weight loss, and anemia.^[11]^ While the patients with normal ESR values in our study had fewer systemic symptoms, there was no significant difference between the groups with and without elevated ESR values in terms of anemia prevalence. Previous studies have revealed similar treatment response, relapse, and GCA development rates to those in the patients in our study who had elevated ESR and/or CRP values.^[10]^ In our study, no GCA or relapse development was observed in the group with normal ESR and CRP values.

The main pre-diagnosis to be considered among patients over the age of 50 who have elevated ESR and CRP values is PMR. In a study in which 201 patients were diagnosed with PMR based on only clinical characteristics and a good response to steroids, 41 patients (20.4%) had ESR values lower than 40 mm/h. No significant difference was found between the group with normal ESR values and the group with elevated ESR values in terms of their clinical characteristics and the course of the disease. The former usually included younger patients with lower rates of fever, weight loss, and anemia.^[11]^ In this study, we determined the rate of ESR < 40 mm/h as 18.5%, the duration of symptoms in these patients was longer, and they had similar ages at diagnosis and rates of systemic symptoms and anemia to those in the ESR ≥ 40 group. Furthermore, the patients in the ESR ≥ 40 group had significantly higher values of CRP and a significantly higher rate of PMR exacerbations.

In another study that was conducted with 460 PMR patients, 7 patients (1.52%) with normal ESR and CRP concentrations at the time of diagnosis were identified.^[5]^ In another study, the rate of normal ESR values at the time of diagnosis was found 6%, while these patients were mostly male, and they had lower CRP values.^[12]^ We found normal ESR values in 27% of our patients, and there was no significant difference between the groups with and without elevated ESR values in terms of their sex distributions. PMR patients can have normal ESR values and less frequently normal CRP values even though they are clinically active.^[13,14]^ In our study, we identified normal CRP values in 9 patients (16.7%) and in this group, like the results in the group of all patients with normal ESR and CRP values, the duration of symptoms was longer, the rates of systemic symptoms and comorbidities were higher, and there were higher ESR values and more PMR exacerbations. Although we did not observe a significant difference in the course of the disease based on the simultaneous presence of normal ESR and CRP values, considering ESR and CRP separately, it may be stated that this group of patients is a group with a milder course of the disease because fewer PMR exacerbations were observed in these patients.

Two small-scale studies were conducted on the potential of ESR values to increase in the follow-up of PMR patients who were diagnosed in the early period and had normal ESR values at the time of diagnosis. While two of the 26 patients who were included in one of these studies showed increased ESR values in the follow-up, none of the 10 patients who were included in the other study showed an increase.^[15,16]^ In our study, none of the patients who had normal ESR and CRP values at the time of diagnosis had increased ESR and/or CRP values in their follow-ups.

In a previous study, APR values were found normal in 62 of 454 PMR patients (14%). It was shown that the duration of PMR symptoms was longer, and the rates of peripheral arthritis and anemia were lower in the patients with normal APR values.^[17]^ In our study, the rate of patients with normal ESR and CRP values was 14%, these patients had longer durations of symptoms, none of them had peripheral arthritis, and there was no significant difference between the group with normal ESR and CRP values and the group with abnormal ESR and/or CRP values in terms of anemia.

As in the case of PMR, ESR values lower than 40 mm/h can be seen in 5% of patients diagnosed with GCA.^[11]^ GCA and PMR are diseases that can overlap with each other and are seen together highly prevalently.^[18]^ The reason why the APR results of some PMR patients are normal can be that they are GCA cases with extracranial vascular involvement and subtle systemic symptoms.^[19]^

There are anecdotal studies on biomarkers other than ESR and CRP in the diagnosis of PMR. It was stated that fibrinogen is as valuable as ESR and CRP in the diagnosis of PMR, and it is even more useful in the assessment of treatment response.^[20]^ Plasma fibrinogen has shown better responsiveness to changes in disease activity and a higher correlation with various patient-reported outcome measures recorded other than ESR and CRP.^[19]^ In a study where the ESR, CRP, and SAA levels of GCA and PMR patients were examined, SAA was determined to be more sensitive than CRP (97% vs 61%, respectively) in determining disease activity and more specific than ESR (86% and 77%) in identifying inactive disease.^[20]^ Another study revealed high SAA levels in PMR patients whose myalgia persisted after tapering steroid treatment despite normal CRP values, and it was stated that SAA can be a beneficial parameter in the evaluation of PMR disease activity.^[21]^ In our study, we analyzed SAA levels in patients with suspected PMR who had normal ESR and CRP values, and we found SAA levels to be high in all of them. The patients in the group with elevated ESR and/or CRP values also had above-normal SAA levels. In our study, the SAA levels of all patients in the group with normal ESR and CRP values at the time of diagnosis were higher than normal, but their values did not differ significantly compared to the values of the group with elevated ERS and/or CRP values.

There may be a few reasons for the normal ESR and CRP values of some patients who were included in our study. The first reason is that these patients could have been misdiagnosed because PMR has a very heterogeneous presentation and no gold standard method for a definitive diagnosis. We used laboratory and imaging methods after evaluating the patients for whom we thought to have PMR with thorough clinical history searching and detailed physical examinations. We included patients who had been followed up for at least 1 year, and we did not observe any other diseases in our patients.

Another reason for the normal ESR and CRP values of these patients may be that they had been diagnosed at an early stage of PMR. However, the fact that the duration of symptoms in the patients with normal ESR and CRP values in our study was longer, and their ESR and CRP values did not increase during follow-up contradicted this possibility. Another hypothesis is that these patients could be GCA cases with extracranial vascular involvement. In our study, no assessment regarding large vessel involvement was made in any patient, and signs and symptoms indicative of large vessel involvement were not observed in their follow-ups. As the final potential reason, these patients could have constituted a subtype of PMR with a milder presentation. When we evaluated the results of the patients separately for ESR and CRP levels that were either elevated or normal, we observed that the rates of PMR exacerbations were lower among those who had normal values compared to those with elevated values. Considering other data in the relevant literature, this seems to be the most feasible scenario.^[17]^

The limitations of this study included its retrospective nature and its limited number of patients, as well as the fact that SAA levels could not be tested in all patients, and USG was not routinely used.

## 5. Conclusion

It was determined that some PMR patients can have normal ESR and CRP values at the time of their diagnosis. In these patients, imaging methods can show findings that support the diagnosis. Additionally, other APR-related tests such as SAA measurements can be utilized. Compared to patients with elevated ESR and CRP values, patients in this group tend to be younger, have fewer systemic symptoms, and have longer durations of symptoms. In cases of patients presenting with typical clinical characteristics of PMR but normal ESR and CRP values, other laboratory and imaging methods should be utilized as supportive steps in the diagnostic process, and the presence of a dramatic response to steroid treatment should be checked.

## Author contributions

**Conceptualization:** Mete Kara, Gülay Alp.

**Data curation:** Mete Kara, Gülay Alp, Ali Murat Koç.

**Formal analysis:** Mete Kara, Ali Murat Koç.

**Investigation:** Mete Kara, Gülay Alp.

**Methodology:** Mete Kara, Gülay Alp, Ali Murat Koç.

**Project administration:** Mete Kara.

**Resources:** Mete Kara, Ali Murat Koç.

**Software:** Mete Kara, Gülay Alp.

**Supervision:** Gülay Alp.

**Validation:** Mete Kara, Gülay Alp, Ali Murat Koç.

**Visualization:** Ali Murat Koç.

**Writing – original draft:** Mete Kara, Gülay Alp.

**Writing – review & editing:** Mete Kara, Gülay Alp.
